# Treatment Outcomes in an Adult Attention Deficit Hyperactivity Disorder Clinic With a Focus on Executive Functioning and Sluggish Cognitive Tempo

**DOI:** 10.7759/cureus.9814

**Published:** 2020-08-17

**Authors:** Sonia Gaur, Stefano Pallanti

**Affiliations:** 1 Psychiatry, Stanford University, Stanford, USA; 2 Psychiatry, Instituto di Neuroscienze, Florence, ITA

**Keywords:** adult adhd, case series, sluggish cognitive tempo, executive function, psychoeducation, modular therapy, self-reports, treatment outcomes, strobe

## Abstract

Aim

Executive function (EF) is considered a core attention deficit hyperactivity disorder (cADHD) symptom in adults and, more recently, sluggish cognitive tempo (SCT). Despite considerable controversy around the role of SCT symptoms in the diagnosis of attention deficit hyperactivity disorder (ADHD), some scholars have suggested that SCT symptoms are a subset of the ADHD syndrome, whereas others have suggested that SCT is an entirely unique type of attention disorder. Therefore, we looked to characterize the impact of treatment as usual (TAU) with medication and psychoeducation on the functional impairments related to EF and SCT, and related functional impairments in adults with ADHD. We aim to clarify if the combination of TAU and modular ADHD therapy (TAUTx) further improves these symptoms. The goal is to assess the validity of self-reporting assessment of symptoms adopted in the present for the monitoring of treatment in this population.

Methods

We implemented the inclusion and exclusion criteria at the onset of the clinic. This prospective cohort case series study is designed to see the difference with self-reporting scales for EF, SCT, and cADHD symptoms in TAU and TAUTx. The STrengthening the Reporting of OBservational studies in Epidemiology (STROBE) checklist was used to provide transparency in reporting the data.

Results

Adults with ADHD showed a significant improvement with TAU in EF (p=0.001), cADHD (p=0.007), and SCT (p=0.002). Furthermore, TAUTx improved areas of EF (p=0.001), cADHD (p=0.004), and SCT (p=0.002). We saw a significant benefit from starting/optimizing medications in the treatment of ADHD along with psychoeducation. Self-reporting scales appeared to be reliable for monitoring the symptoms of ADHD and related dysfunction and were consistent with the Clinical Global Impressions Scale.

Conclusions

Adult ADHD patients reported significant benefit from TAU for aspects of impairment in EF and SCT. They require ongoing medication prescribing and “tailoring” through optimization. Psychoeducation is an effective form of therapy in these patients with or without the addition of adult ADHD modular therapy. Self-reporting is valid for monitoring and providing transparency in patient care.

## Introduction

Background/rationale

The prevalence of adult attention deficit hyperactivity disorder (ADHD) can be 2.9-16.4% [1] or 15.8-17.4% [2], depending on studies. ADHD is a disorder characterized by deficits in the attention span (inattention), impulsive behaviors (impulsivity), and increased level of activity (hyperactivity) [3]. ADHD can cause significant impairment in areas of cognitive and executive functions (EF). Furthermore, the behavioral problems that result from untreated ADHD can affect personal and social relationships, academic functioning, and work performance, leading to a lower quality of life [2]. The lifetime prevalence of having comorbidity is 60-80% [4], which confounds the diagnoses and complicates the treatment of ADHD [5].

Standardized screening can assess the functional aspects of ADHD and their comorbidities. These screening tools can monitor and assess the baseline of parameters that can contribute to the impairment in ADHD. Though the functional aspects include EF and ADHD scores, one of the known confounders in the diagnoses of ADHD in children is sluggish cognitive tempo (SCT) [6], which refers to a clinical construct of sluggishness, absent-mindedness, and low energy.

While some scholars have suggested that SCT symptoms are a subset of the ADHD syndrome [7-8], others have suggested that SCT is an entirely distinct type of attention disorder [6]. SCT symptoms appear to be separable from ADHD symptoms of inattention and hyperactivity as noted in factor analytic studies [9]. Commonly associated with the inattentive type of ADHD, the benefits of ADHD treatment for SCT patients is still unclear. Of those who are on stimulants, there was a relationship between SCT and EF [10]. Designing a prospective treatment outcome study for these functional aspects of impairments may guide practitioners on prescribing and optimization of medications. It would also help guide areas of further interest.

It is not uncommon to find monitoring of the treatment of children and adolescents with ADHD in academic and community settings. However, it is rare in the treatment of adults with ADHD. Studies with long-term treatment outcomes in adults with ADHD have shown limited conclusions but are in favor of treatment rather than non-treatment [11-12]. The results of the response to treatment of the ADHD symptoms in a clinically referred sample of children showed an association between EF and SCT [13-14].

## Materials and methods

Methods

On entry to the clinic, all participants underwent a self-reported narrative psychiatric assessment and a structured or semi-structured standardized interview for ADHD: DIVA (Diagnostic Interview for ADHD in adults) 2.0 [15] and ACE (ADHD Child Evaluation) plus [16]. Inter-rater reliability between a child psychiatrist (first author) and an adult psychiatrist (second author) who came from different clinical backgrounds was 100% for the psychiatric and structured assessment for the same interview and 90% for a different patient seen on two visits. The primary author treated all the participants and subjects.

Validity and cost-effective were part of the decision-making in choosing self-reporting forms for adults with ADHD. Forms used for the functional impairments of EF, core ADHD (cADHD), and sluggish cognitive were Barkley Deficits in Executive Function-Scale (BDEFS-LA) [17] and Barkley Adult ADHD Rating Scales version IV (BAARS-IV) [18]. Self-reports are valid for the diagnosis of ADHD [19], as opposed to multi-informant reporting in the evaluation of child and adolescent ADHD [20]. Neuropsychological tests showed low to no relationship between impairments in various domains of major life activities and ratings of EF symptoms in daily life in those adults (low ecological validity) [21]. Of note, a popular neuropsychological test, the continuous performance test (CPT), showed no evidence of the clinical utility of the CPT to assess or monitor ADHD in adults [22]. There is no evidence in the literature comparing the adult modular therapies [23-25] and the choice was to use the Safren manual, as we consider it a gold standard in some professional circles.

The study design compared the groups using self-rating scales of different functional impairments seen in ADHD. The BDEFS-LA subscales were as follows: self-management to time, self-organization/problem solving, self-motivation, self-regulation of emotions, total EF, and ADHD-EF index. The last index subscale shows the likelihood that the respondent has ADHD. The BAARS-IV subscales were as follows: inattention, hyperactivity, impulsivity, total scores, symptom scores, and SCT. Manuals published by both BDEFS and BAARS had data for the Reliable Change Index (RCI), which was referenced for these participants.. The Adult ADHD Self-Report scale version 1.1 was used to monitor weekly progress during the therapy.

Recruitment of subjects was stopped in October 2019. The two groups were as follows: group I/TAUTx with 14 subjects who received the treatment arm of medication, psychoeducation (PE), and adult ADHD therapy (Tx), and group II/TAU with 18 subjects who received medication initiation/optimization and PE. Self-reports at baseline entry into the clinic, at medication optimization with PE, and at the end of Tx were collected. Data were collected of the known variables in ADHD, such as substance use, sleep disorders, medical and psychiatric contributors, and the age of onset, to provide further explanations of the results.

Inclusion and exclusion criteria

The inclusion criteria were a diagnosis of ADHD and 18 years of age or above. The exclusion criteria were active mood disorder, suicide ideation, and schizophrenia spectrum. Substance use disorder was part of the exclusion criteria for all therapy cases, but its minimal use with efforts to decrease/abstain was included in the medication and PE group.

Statistical methods

All data analysis was performed using IBM SPSS software, Version 25 (IBM Corp., Armonk, USA). The Wilcoxon signed-rank test [26] was used for the analysis of the data. The Wilcoxon signed-rank test is the nonparametric test equivalent to the dependent t-test. The test does not assume normality in the data and is used when this assumption is violated or the use of the dependent t-test is inappropriate. It is used to compare two sets of scores that come from the same participants. This can occur when we wish to investigate any change in scores from one time point to another or when individuals are subjected to more than one condition.

Setting

Participants aged 18-70 years who were referred to and comprehensively evaluated at a specialty service for ADHD at a tertiary referral medical center in an urban setting were included. The referrals to the clinic came from community primary care clinics through a standard intake process prior to assignment to the specialty clinics at the tertiary center from September 2018 to February 2020. The clinic saw 113 patients during that period. All patients were asked to fill out the self-report forms. There was a waitlist created for the patients interested in engaging in therapy and the first-come base criterion was used. The recruitment was ongoing and discontinued in October 2019. This was a consultation and treatment model clinic and all subjects were regularly followed up. The clinic was discontinued in February of 2020 with a three-month notice and a referral to community caregivers to all participants. The primary author collected data at entry to the clinic, three to six months after the first entry into the clinic, and at the end of therapy. IRB approval for a chart review was obtained.

**Figure 1:**
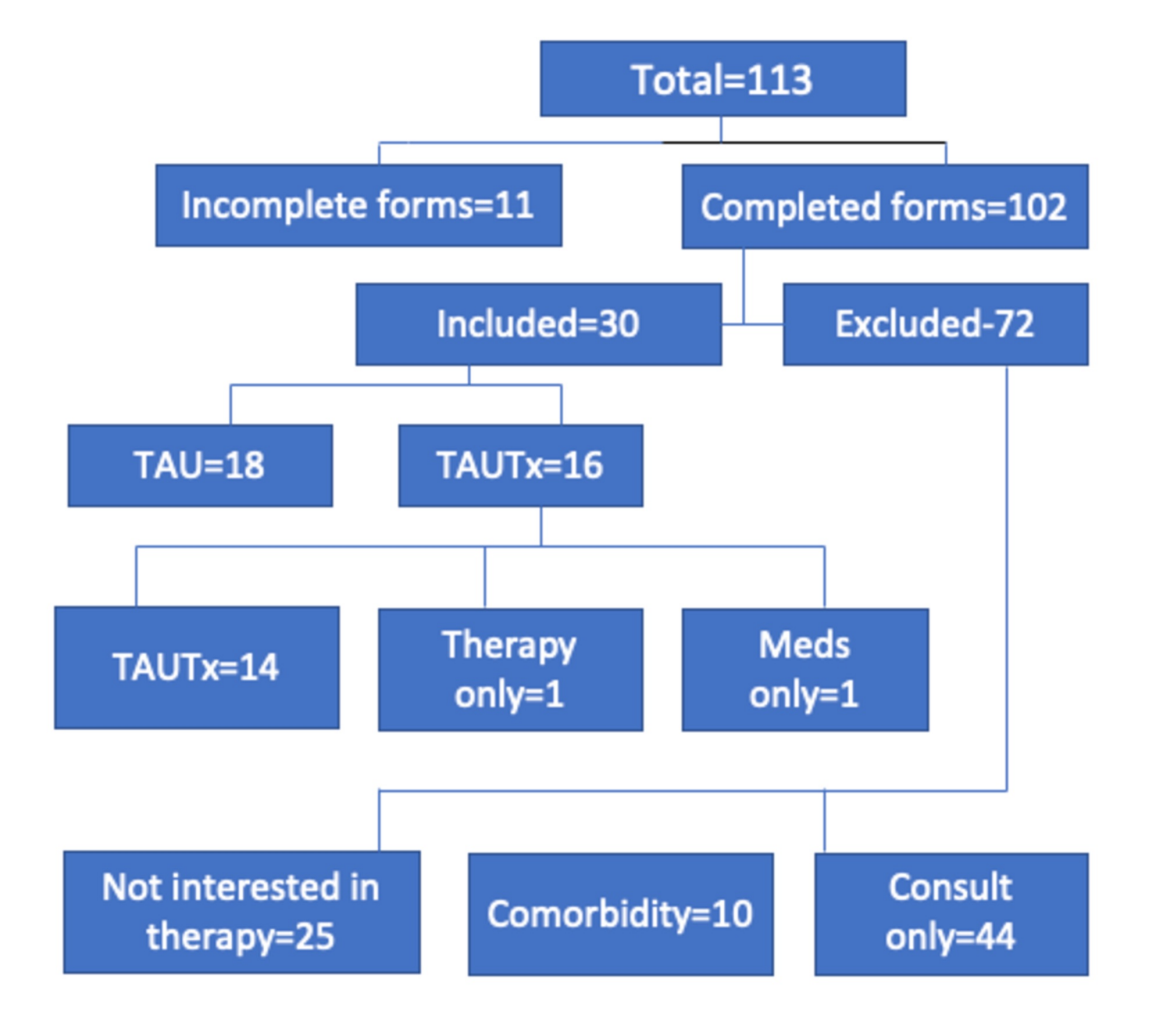
Flow diagram of participants TAUTx, medication management and modular adult attention deficit hyperactivity disorder therapy; TAU, medication management and psychoeducation

Figure 1 shows the total number of participants we drew the subjects from as well as breakdown of the recruitment process. A total of 113 participants enrolled in the clinic. Out of those, 25 were not interested in engaging in the therapy and 44 were only interested in a consultation for their ADHD. Comorbidity in 10 of them lead to their exclusion in the study. Though most of them completed the BDEFS/BAARS, 72 out of 102 did not take part for several reasons, of whom the majority (95.8%) were not interested in ongoing care/therapy.

Demographics of participants

Table 1 shows the breakdown of age, gender, race/ethnicity, education, employment, and intelligent quotient as variables in the subjects.

**Table 1:** Demographics of subjects IQ, intelligence quotient

Category	Total number of subjects = 28
Gender	
Male	18
Female	10
Race/ethnicity	
Caucasian/White	17
South Asian	2
Asian	4
Hispanic/Latino	2
Middle Eastern	3
Ages included as a range	26-57
Education	
College degree and/or plus	27
High school graduate and/or technical school	1
Employment	
Yes	27
No	1
IQ more than 120	13
Primary education outside the USA	14

## Results

Table 2 shows the different group comparisons of pre- and post-functional constructs using the scores from the BDEFS-LA and BAARS-IV. It shows the areas of total EF, SCT, total ADHD scores, EF scores, and symptoms. Adults with ADHD benefit from medication and psychoeducation in areas that affect EF (p=0.001), cADHD (p=0.007), and SCT (p=0.002). The addition of modular ADHD therapy helps areas of EF (p=0.001), cADHD (p=0.004), and SCT (p=0.002). We saw significant improvement from starting and optimizing medications in the treatment of ADHD along with psychoeducation and/or modular therapy.

**Table 2:** Results of executive functioning and sluggish cognitive tempo ADHD, attention deficit hyperactivity disorder; BDEFS, Barkley Deficits in Executive Function Scale; Barkley Adult ADHD Rating Scales; BAARS; N. ranks, negative ranks, P. ranks, positive ranks; Asymp. Sig.: asymptotic significance; TAU, treatment as usual: medication and psychoeducation; TAUTx, treatment as usual: medication, psychoeducation, and modular adult therapy

Group	Wilcoxon signed-rank test	Total executive function (BDEFS)	Total ADHD score (BAARS)	Sluggish cognitive tempo (BAARS)	Executive function index (BDEFS)	Executive functions symptoms (BDEFS)
Group I/TAUTx	N. ranks	14	12	12	13	13
	P. ranks	0	2	0	1	1
	Ties	0	0	2	0	0
	Total	14	14	14	14	14
	Z score	-3.296	-2.858	-3.076	-3.126	-3.234
	Asymp. Sig. (two-tailed)	0.001	0.004	0.002	0.002	0.001
Group II/TAU	N. ranks	16	16	13	12	16
	P. ranks	2	2	2	6	2
	Ties	0	0	3	0	0
	Total	18	18	18	18	18
	Z score	-3.310	-2.701	-3.087	-1.810	-3.050
	Asymp. Sig. (two-tailed)	0.001	0.007	0.002	0.070	0.002

Normalization and reliable change index as published for these forms (BDEFS-LA/BAARS-IV) showed a normalization of symptoms for most of the subjects in areas of EF, cADHD, and SCT. Of the group that had reliability scores consistent with published data (N=14), 100% of them had a difference of more than 54 points in EF and 71% had had a difference of more than 2 points in SCT. In the group that had unreliability scores consistent with published data (N=10), the difference in scores was less than 32 points in EF and less than 2 in the SCT group. Of the subjects that showed the unreliable scores, 80% of them were on three different medications.

**Figure 2:**
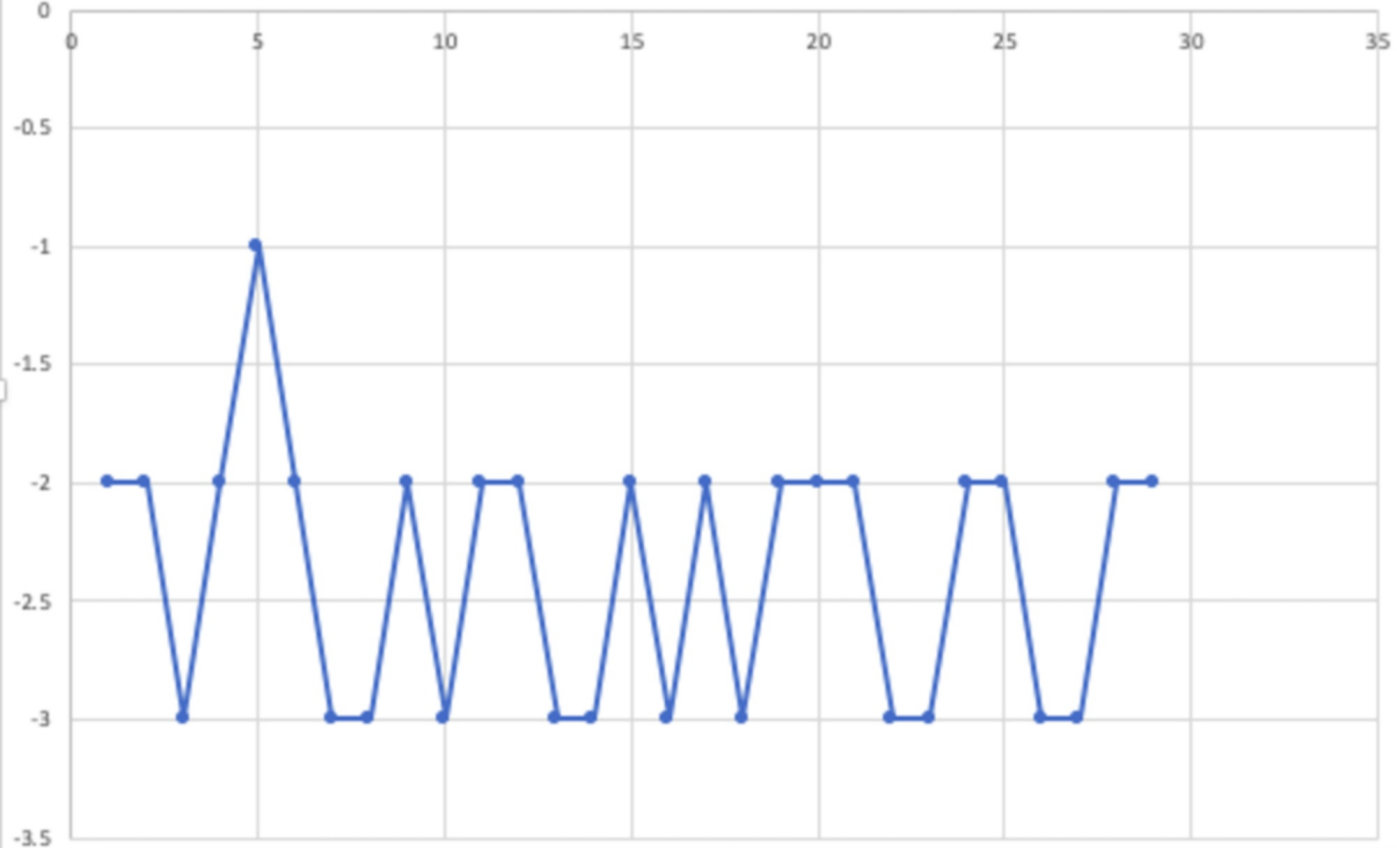
Difference in CGI scores pre- and post-treatment of TAU and TAUTx groups The X-axis shows the identification number assigned to each subject. The Y-axis shows the difference between pre- and post-treatment in CGI scores, and all of them showed an improvement in scores. TAU, treatment as usual: medication and psychoeducation; TAUTx, treatment as usual: medication, psychoeducation, and modular adult therapy

The Clinical Global Impressions Scale (CGI) pre- and post-score differences are shown in Figure 2. Higher incidence of medical comorbidity, obsessive-compulsive disorder, substance use disorder, and engaging in therapy without medications correlated with a poor outcome. The graph shows the difference between pre- and post-treatment. It produced an improvement of ADHD core symptoms, including EF and SCT, and this is consistent with the validity of the clinical construct of these symptoms and the validity of the subjective response that correlated with the aim of clinical improvement.

Sleep disorders were common (69%) in this cohort. Out of those, the sleep disorders were obstructive sleep apnea (14%), initial insomnia (52%), initial and middle insomnia (17%), and narcolepsy (3%). Substance abuse disorders in the past of 48% remitted to 3% with treatment. Of those who used substances, marihuana/cannabinoids versus alcohol was their drug of choice by 58% and 42%, respectively.

The results were consistent for both genders in the group that underwent ADHD therapy. The subjective reporting of improvement from the therapy was greater in males than females. Among the females, 14.2% reported minimal benefit in follow-up notes but reported functional improvement that was statistically significant in the self-report. All of them reported subjectively, with minimal to no positive effect from the module (distractibility delay), with 86.7% reporting subjective improvement with the addition of adult ADHD therapy.

**Figure 3:**
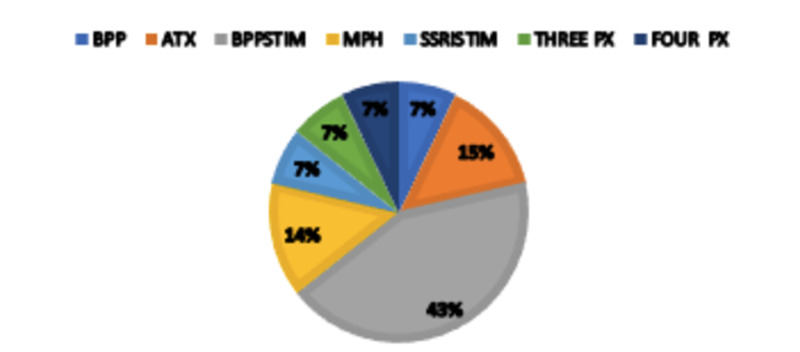
Medications types used in the TAU group by percentages TAU, treatment as usual: medication management and psychoeducation; BPP, bupropion; ATX, atomoxetine; BPPSTIM: bupropion and stimulant; MPH, methylphenidate; SSRISTIM, selective serotonergic reuptake inhibitor and stimulant; THREE PX, three classes of psychotropics; FOUR PX, four classes of psychotropics

**Figure 4:**
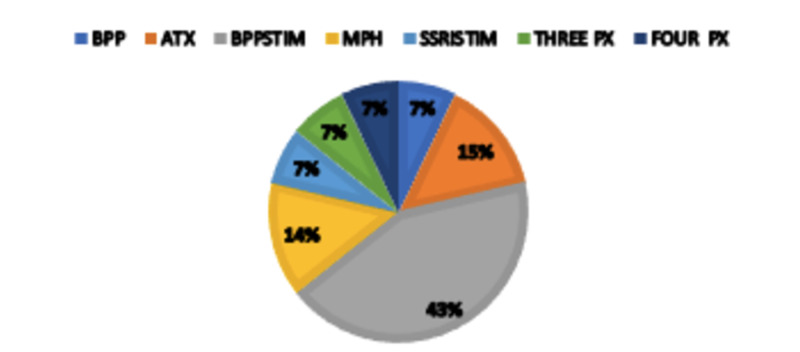
Medications types used in the TAUTx group by percentages TAUTx, treatment as usual: medication, psychoeducation, and modular adult therapy; BPP, bupropion; ATX, atomoxetine; BPPSTIM: bupropion and stimulant; MPH, methylphenidate; SSRISTIM, selective serotonergic reuptake inhibitor and stimulant; THREE PX, three classes of psychotropics; FOUR PX, four classes of psychotropics

Figures 3, 4 show two-dimensional pie graphs that demonstrate comparison between groups in terms of the medication choice for TAU and TAUTx. The subjects who underwent therapy have a preference for the combination of atomoxetine and a stimulant (29% vs. 0%), whereas those in the TAU group were remarkable in using the bupropion and stimulant combination (43 vs. 7%). Both groups were similar in their use of monotherapy as well as combinations of three or four classes of psychotropic medications for treatment. Bupropion was not used alone as a treatment.

## Discussion

To the best of our knowledge, this is the first time a study has shown that self-reporting of SCT and EF is associated with each other and improves with medication and psychoeducation. Medication initiation/optimization with psychoeducation with or without modular ADHD therapy improves the symptoms and the functional aspect of all the symptoms of subjects recruited for EF and SCT. The results of the response to treatment of EF and SCT in a clinically referred sample of adults with ADHD supported an association between core symptoms, EF and SCT, and treatment [10].

Self-report rating scales, as reported previously, are reliable and valid assessments in the treatment response and monitoring of patients [19]. Modular adult ADHD therapy is beneficial in all aspects of ADHD [27], but the subjective response to the module of distractibility shows scarce benefit compared to the other modules. Moreover, psychoeducation appeared effective as therapy to improve the symptoms of ADHD without modular adult ADHD therapy.

The difference in treatment response is remarkable when we looked at medication in combination, such as bupropion versus atomoxetine in TAU versus TAUTx group (42.9% vs. 7.1% and 29% vs. 0%, respectively). Studies on bupropion have inconsistent results, but the studies have been small [28], and there is no evidence in the literature on using bupropion in combination with a stimulant to treat adult ADHD. Looking at combination studies may help us understand this further. Interpretation of the normalization and reliable change in BDEFS-LA/BAARS-IV data in conjunction with the clinical picture is supported by this study.. Using ranges of scores to reestablish difference, EF difference can be between 32 and 54 and SCT difference can be 2 or more. Perhaps future studies targeting meaningful data, multiple, or repeat assessment would help describe the trajectory of the change. The co-relation of sleep disorders (60-80%) is not explained by factors such as use of stimulants and types of ADHD [29], which was seen in this study. The clinical constructs of SCT [6-8] and sleep disorders can mimic each other, but our study numbers were small to make an interpretation; a higher number of subjects using objective measures of SCT may give more meaningful results. Remission of substance abuse disorders can change with the treatment of ADHD, as seen in previous studies. [4,5]. Education around sleep and substance abuse showed a positive trend and may become more meaningful with a larger sample size.

Observational psychiatric studies can be meaningful if there is a discussion of bias to provide transparency. Awareness and attempts to eliminate it are more meaningful in presenting data [30]. This study is designed in a treatment clinic and is a naturalistic report of clinical activities in an adult ADHD outpatient clinic.. Preference for a structured interview DIVA 2.0 rather than a semi-structured ACE PLUS created a variable in the diagnosis of ADHD. Future data can look at comparing the two interviews in consistency for the diagnosis and type of ADHD. Among the three modular components in the adult ADHD therapy, all subjects reported no benefit from the distractibility module. More research in this module may be helpful.

Inter-rater reliability was 90-100%, and collateral information when available was obtained. This prevented confounders in diagnosis. Choosing evidence-based modular therapy facilitated the decrease in subjective and the variables in the relationship between the therapist and unconscious bias. However, this bias is helpful in translating it to real life practice. It was a conscious effort to include equal amounts of males and females but did not include the transgender population, as they did not take part in the therapy. Understanding why this population did not take part may help us understand if cross-cultural bias was present. The subjects had a college education and employment at the time of the study. This does not represent the people who did not complete their education or the unemployed. Substance use was an exclusion criterion for therapy, which may not generalize to the population.

Adult ADHD is commonly treated in the community by an adult psychiatrist or a family physician, but in this study an experienced child and adolescent psychiatrist treated them. Medications are not tolerated by all individuals [28], and therefore therapy can be important for treatment to ameliorate symptoms. Psychoeducation as a therapeutic tool varies depending on the background, training, and experience of the provider. Future studies looking at standardization of PE in adults with ADHD may be helpful.

## Conclusions

In conclusion, medication initiation and optimization help EF, SCT, and cADHD symptoms that impair adults with ADHD. Given our initial specific aims, the most noteworthy finding to emerge from this study is the significant relationship between the response to the treatment of the SCT and EF.

 The nature of the responses to the treatment is consistent with the hypothesis that there is a close correlation between these different dimensions. These findings can help the physician be less hesitant to prescribe or continue medications. It may help set standards for treatment recommendations for psychoeducation and medication monitoring and may improve the distractibility delay module of therapy.
